# Integrated analysis of the methylome and transcriptome of chickens with fatty liver hemorrhagic syndrome

**DOI:** 10.1186/s12864-020-07305-3

**Published:** 2021-01-06

**Authors:** Xiaodong Tan, Ranran Liu, Yonghong Zhang, Xicai Wang, Jie Wang, Hailong Wang, Guiping Zhao, Maiqing Zheng, Jie Wen

**Affiliations:** 1 State Key Laboratory of Animal Nutrition, Institute of Animal Sciences, Chinese Academy of Agricultural Sciences, Beijing, 100193 China; 2 College of Animal Science, Jilin University, Changchun, 130062 China

**Keywords:** DNA methylation, RNA-seq, Fatty liver hemorrhagic syndrome, Lipid metabolism, Cellular junction and communication

## Abstract

**Background:**

DNA methylation, a biochemical modification of cytosine, has an important role in lipid metabolism. Fatty liver hemorrhagic syndrome (FLHS) is a serious disease and is tightly linked to lipid homeostasis. Herein, we compared the methylome and transcriptome of chickens with and without FLHS.

**Results:**

We found genome-wide dysregulated DNA methylation pattern in which regions up- and down-stream of gene body were hypo-methylated in chickens with FLHS. A total of 4155 differentially methylated genes and 1389 differentially expressed genes were identified. Genes were focused when a negative relationship between mRNA expression and DNA methylation in promoter and gene body were detected. Based on pathway enrichment analysis, we found expression of genes related to lipogenesis and oxygenolysis (e.g., PPAR signaling pathway, fatty acid biosynthesis, and fatty acid elongation) to be up-regulated with associated down-regulated DNA methylation. In contrast, genes related to cellular junction and communication pathways (e.g., vascular smooth muscle contraction, phosphatidylinositol signaling system, and gap junction) were inhibited and with associated up-regulation of DNA methylation.

**Conclusions:**

In the current study, we provide a genome-wide scale landscape of DNA methylation and gene expression. The hepatic hypo-methylation feature has been identified with FLHS chickens. By integrated analysis, the results strongly suggest that increased lipid accumulation and hepatocyte rupture are central pathways that are regulated by DNA methylation in chickens with FLHS.

**Supplementary Information:**

The online version contains supplementary material available at 10.1186/s12864-020-07305-3.

## Background

For chickens, fatty liver hemorrhagic syndrome (FLHS) is a serious disease, which is characterized by massive lipid accretion and hemorrhagic spots of the liver [1]. The prevalence of FLHS has been reported to be 4% and even up to 16% [2, 3], especially for native chickens. The physiological characteristics of FLHS are quite different from common fatty liver syndrome (FLS). FLHS is more serious than FLS due to the severe rupture of hepatocytes and blood vessels with conspicuous hemorrhagic liver spots. For standard chicken farming, FLHS accounts for 28 to 74% of all mortality [4, 5].

FLHS is tightly linked to lipid homeostasis, with disorders of synthesis, transport, and oxygenolysis [6]. Individuals with FLHS have elevated lipid metabolism, dominated by an anabolic process. With increased triglyceride (TG) deposition in hepatocytes, enlarged hepatocytes and histological injury are observed [7]. Moreover, the disappeared cellular boundaries and destroyed cellular junction are discovered, and the impaired hepatocyte structure is observed distinctly [8, 9].

Both environmental and genetic factors contribute to FLHS with possibility for involvement of epigenetic modifications [10, 11]. In particular, DNA methylation, an important epigenetic modification, has been closely associated with hepatic lipogenesis and fatty liver [12, 13]. It is widely recognized that transcriptional activation is inversely correlated with DNA methylation [14]. Therefore, an integrative analysis of both the transcriptome and the methylome is necessary for a full understanding of the involvement of epigenetics in FLHS. Previous reports have demonstrated lipid metabolism to be up-regulated with differential gene methylation in individuals with fatty liver disease [15]. Liu et al. identified lipid metabolism genes (*ACACA* and *MTTP*) to be up-regulated due to alterations in DNA methylation [16]. Sookoian et al. demonstrated hyper methylation of *PPARγ* in fatty liver subjects [17].

In previous study, Zhang et al. described a chicken FLHS model [3]. However, they did not evaluate the role of DNA methylation in the model. Herein, the aim of this study was to perform an integrated analysis of this FLHS model by examining the DNA methylome and transcriptome of chickens with FLHS.

## Results

### Comparison of DNA methylome profiles of chickens with and without FLHS

**Fig. 1:**
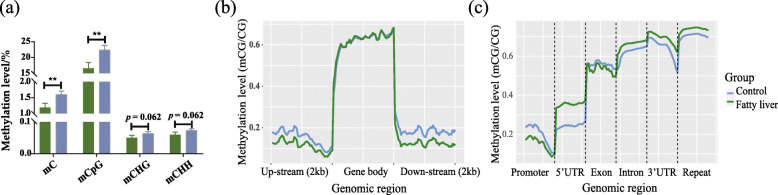
Global methylation pattern in normal and liver and fatty liver. **a** Genome-wide methylation level in two groups. **b** Distribution of methylation in gene body, up-stream and down-stream. Gene body, from TSS to TES; Up-stream (2 kb), 2000 bp of upstream region from TSS; Down-stream (2 kb), 2000 bp of downstream region from TES. **c** Distribution of methylation in various regions. Including promoter, 5’UTR, 3’UTR, intron, exon and repeat region

Hepatic DNA methylomes of chickens with and without FLHS were compared to determine whether hepatic lipid metabolism was regulated by methylation changes. Overt lower genome-wide methylation levels were detected in the fatty liver group (Fig. 1a). The hepatic CpG (C represents cytosine and G represents guanine, while p represents phosphate bond between nucleotides) methylation levels of FLHS were lower in regions up- and down-stream of gene bodies, while it’s not identical to that in the gene body, the methylation difference in the gene body was relative small (Fig. 1b). Methylation levels of various functional regions around the gene body were assessed and found to be decreased in promoter and exon regions, but elevated in 5’UTR, intron, 3’UTR, and repeat region (Fig. 1c). Similar methylation alterations were detected in CHG and CHH sites (H represents A, C, or T) (Supplementary Figure S2).

### Identification and distribution of differentially methylated regions (DMRs)

**Fig. 2:**
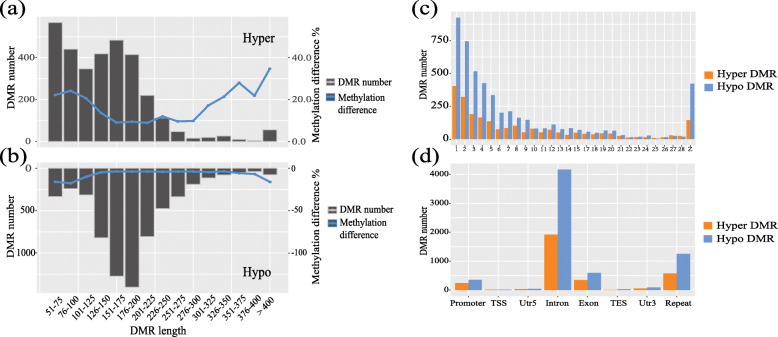
Statistic for DMR in genome-wide scale. **a** The count and methylation difference of hyper DMR with various length. The x-axis indicates the DMR length, the left and right y-axis indicates the number and methylation difference of DMR with various length. **b** The count and methylation difference of hypo DMR with various length. **c** Statistic for hyper and hypo DMR count in each chromosome. **d** Statistic for hyper and hypo DMR in various regions. Including promoter, TSS, 5′ and 3′ UTR, intron, exon, TES, and repeat region

A total of 7623 DMRs were identified between the two groups. The length of DMRs ranged from 51 bp to more than 400 bp, with most DMRs centered on limits of 50 bp to 200 bp. Absolute methylation difference was under 40% (Fig. 2a-b). Chromosome distribution of DMRs is shown in Fig. 2c, with the number of DMRs in various functional region enumerated (Fig. 2d). Most enrichment was in intron, with little enrichment in TSS, 5’UTR, 3’UTR, or TES regions.

### Global gene methylation profile and differentially methylated genes (DMGs) detection

**Fig. 3:**
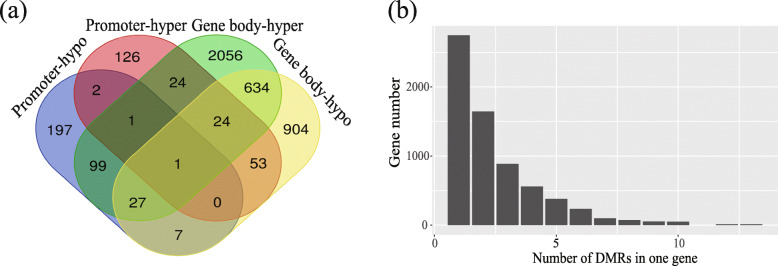
Methylation pattern and DMR number within DMGs. **a** Overlapping for DMGs with multiple DMRs. **b** Statistic for DMGs number with different number of DMRs

We defined the DMGs when DMRs overlapped with annotated genes. A total of 4155 DMGs were detected (Supplementary Table S1). Among which 561 DMGs were identified as DMRs in promoter regions including 227 hyper-methylated DMGs and 330 hypo-methylated DMGs, with four DMGs identified as both hyper- and hypo-methylated DMRs in promoter regions (Fig. 3a). Likewise, 3830 DMGs were identified as DMRs in gene body regions including 964 hyper-methylated DMGs and 2180 hypo-methylated DMGs, with 686 DMGs identified as both hyper- and hypo-methylated DMRs in gene body regions (Fig. 3a). The number of DMGs with various DMR number are shown in Fig. 3b. Most DMGs possessed less than three DMRs within corresponding regions.

### Integrative analysis of differentially expressed genes (DEGs) and DMGs

**Fig. 4:**
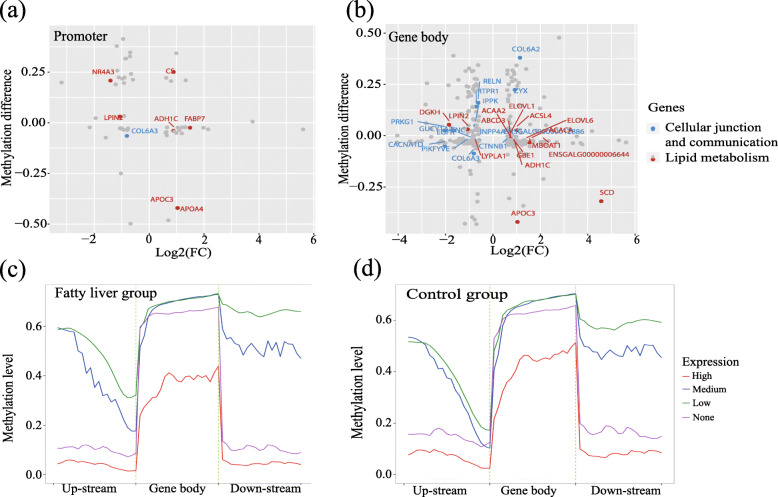
Overlapping genes between DMGs and DEGs. **a** Methylation and transcription levels of overlapping genes with DMR in promoter region, red point indicates the lipid related genes while blue point indicates the cellular junction and communication related genes. **b** Methylation and transcription levels of overlapping genes with DMR in gene body region. **c** The methylation levels of all genes with different transcriptional levels in gene body, up- and down-stream in fatty liver group. **d** The methylation levels of all genes grouped by transcriptional levels in gene body, up- and down-stream in control group

The role of DNA methylation in mRNA expression was explored by integrative analysis of whole-genome bisulfite sequencing (WGBS) and RNA-seq. A total of 1389 DEGs were defined (Supplementary Table S2), of which 318 overlapped with DMGs (Supplementary Table S3). In addition, some of the overlapping genes were annotated to be closely linked to lipid metabolism and transport (e.g., *ACACA*, *APOA4*, and *SCD*) as well as cellular junction and communication (e.g., *PRKG1*, *ITPR1*, and *DGKH*) (Fig. 4a-b), which suggests an important role for DNA methylation in lipid homeostasis and hepatocyte structure. In promoter regions (2 kb up-stream of gene bodies), methylation levels were negatively correlated with gene expression. Methylation levels were stable for genes with high and no expression, with a decreasing tendency for regions up-stream of the TSS site of the genes with low and medium expression (Fig. 4a-b, Supplementary Figure S3). In down-stream regulatory regions, specific and stable methylation was found for genes with various expression levels (Fig. 4c-d, Supplementary Figure S3). In the gene body, the methylation levels were similar to those in down-stream regions, with higher levels compared to the up- and down-stream regulatory regions.

### Pathway enrichment analysis of overlapping genes

**Fig. 5:**
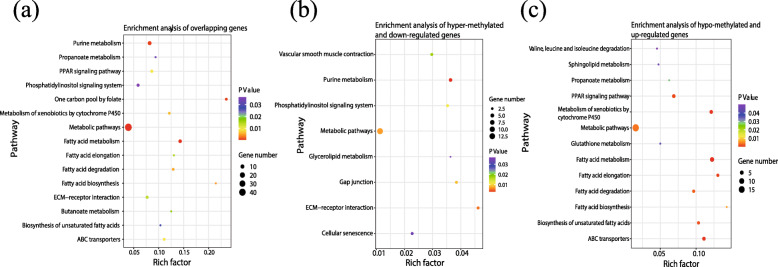
Pathway enrichment analysis of overlapping genes. **a**-**c** Pathway enrichment result with all the overlapping genes, hyper-methylated and down-regulated genes, and hypo-methylated and up-regulated genes, respectively

KEGG enrichment analysis was performed with the overlapping genes of DMGs and DEGs. For all overlapping genes, six of 14 pathways directly related to lipid metabolism were significantly enriched. Some of the key genes of lipid metabolism enriched in those pathways were *ACACA*, *SCD*, and *APOC3*, as well as other overlapping genes. In addition, the phosphatidylinositol signaling pathway, related to cellular communication, was also found to be significantly enriched (Fig. 5a). For overlapping hyper-methylated DMGs and down-regulated DEGs, glycerolipid metabolism was significantly enriched, which indicates a reduced synthesis of diacylglycerol. Gap junction and the phosphatidylinositol signaling pathway were down-regulated. Both are related to cellular junction and communication and included *ITPR1*, *PRKG1*, and *IPPK*, as well as other genes (Fig. 5b). For overlapping of hypo-methylated DMGs and up-regulated DEGs, 13 pathways were significantly enriched, which indicates the activation of lipogenesis and oxygenolysis (e.g., PPAR signaling pathway, fatty acid metabolism, and fatty acid biosynthesis) (Fig. 5c).

## Discussion

FLHS is distinguishable from FLS in chickens based on hemorrhagic symptoms. Both FLHS and FLS feature by excessive lipid accumulation [9]. With lipid deposition and no treatment, mild FLS develops in to severe FLHS. In previous studies, we reported an induction method and reproduction mode by which to generate a fatty liver chicken line [3]. For successive generations of the line, fatty liver becomes less severe and presents only hepatocyte steatosis rather than a hemorrhagic phenotype. We previously compared the epigenetic features of chickens with mild FLS rather than severe FLHS [15]. Herein, we compared the methylome and transcriptome of chickens with and without FLHS, to identify the effect of DNA methylation on regulatory pathways during FLHS.

The analysis of epigenetic modifications is widely regarded as a valid approach to investigate the molecular basis for a syndrome [18]. DNA methylation analysis is a common approach and has been shown to play a crucial role in the development of fatty liver [19]. In a previous study, we reported lower methylation levels in gene bodies, which included up- and down-stream regions [15]. Our results here are similar to those of our previous study, in that lower methylation levels were also identified in regulatory regions (up- and down-stream of the gene body). These results suggest comprehensive alterations of the gene expression profile, indicating a global effect on the FLHS methylome. A similar methylation profile was reported by the non-alcoholic steatohepatitis (NASH) study, with 76% hypo-methylated and 24% hyper-methylated CpG sites in patients suffering advanced NASH compared to mild NASH [13]. This is consistent with our findings, and suggests that distinct characteristics of the methylome may be useful for diagnosing fatty liver.

Although the relationship among DNA methylation and gene expression is quite complex and difficult to fully explain, DNA methylation is often considered a mechanism for transcriptional repression [20]. In this study, four DEG groups with none, low, medium, and high gene expression levels were generated and the average methylation level of those genes in each group was calculated and compared correspondingly (Fig. 4c-d). This approach could show the correlation of DNA methylation and gene expression globally, although no typical correlation coefficient was calculated and provided [21]. A negative correlation was found for genome-wide methylation and gene expression. Within promoter regions, a decreasing methylation trend close to the TSS site was observed for genes with medium and low expression level, which is consistent with transcriptional activation. For highly expressed genes, loss of methylation may result in elevated expression levels [21]. For a fine and effective methylation map of fatty liver, a smoothing method was applied to detect DMRs highly associated with metabolic syndrome [22, 23]. A total of 7623 DMRs were identified with lengths between 50 bp and 200 bp, with DMR length approaching a normal distribution [24].

DMGs were identified by the overlap between functional genes and DMRs. A total of 4155 DMGs were found between the two groups based on transcriptional profile, with 318 overlapping genes between DMGs and DEGs identified. For these, genes related to lipid metabolism had increased expression levels and hypo-methylated DMRs. ACACA, a key enzyme of de novo lipogenesis [25], was hypo-methylated with up-regulated expression. In fatty liver chickens, pathways of lipogenesis were found to be substantially elevated, with similar alterations of both methylation and expression as previously reported [16, 26]. *APOA4* has been tightly linked to hepatic triglyceride export into serum [27]. Kim et al. reported a negative correlation between DNA methylation and gene expression of *APOA4* in fatty liver individuals [28], which is consistent with our results. Likewise, *SCD*, *ELOVL6*, and *APOC3* were found to have an alteration in both DNA methylation and gene expression. Each of these genes could be a target gene regulated by epigenetic modification in the process of FLHS. Genes related to cellular junction and communication were found to have hyper-methylated DMRs and decreased expression levels. *PRKG1* is involved in the gap junction pathway and is related to metabolic syndromes. Hong et al. demonstrated *PRKG1* to be hypo-methylated and increasingly expressed in a fatty liver model induced with oleic acid [29]. Differences in that study from ours may be due to different pathological processes resultant form differences in the methods of induction. Rachel et al. demonstrated *ITPR1* specific knock-out mice could reverse fatty liver [30], although methylation data were not provided. We found *ITPR1* to be involved in cellular junction and communication pathways, with hyper-methylated DMRs and lower expression levels. These results are slightly different from previous reports and may be due to differences in animal models. Our model is more influenced by the rupture of hepatocytes and vessels with genes down-regulation.

Due to the comprehensive nature of the relationship between DNA methylation and gene expression, involved pathways were enriched with the common genes described above. For hyper-methylated and down-regulated genes, most were enriched in the cellular junction and communication pathways (e.g., gap junction, phosphatidylinositol signaling system, and vascular smooth muscle contraction). Manuel et al. demonstrated that impaired intercellular communication and gap junction were involved in the fatty liver pathological process, with gap junction playing a protective role by maintenance of homeostasis through cell-to-cell communication [31]. Reduced glycerolipid metabolism indicates decreased synthesis of diacylglycerol, which serves as a second messenger for cell signal transduction [32], in conjunction with the phosphatidylinositol signaling pathway. We identified blocked phosphatidylinositol signaling transduction as well as dysfunction of the synthesis of diacylglycerol by FLHS individuals, indicating impaired signaling transduction in hepatocytes. The result is an accumulation of TG, hepatocyte rupture, and hemorrhagic spots. Broken hepatocyte and blood vessel structure could account for the dysfunction of cellular junction and communication pathways as well as vascular contraction. We found these pathways to be regulated by DNA methylation, which implies that hepatocyte rupture and a hemorrhagic phenotype are regulated by DNA methylation.

Hypo-methylated and up-regulated genes related to fatty acid metabolism were involved in biosynthesis and elongation of fatty acids, as well as PPAR signaling pathways. The PPAR signaling pathway is widely regarded as a hub target for lipid metabolism, the inhibition of which could dampen hepatic fat accumulation, relieving fatty liver [33]. Sookoian et al. found hyper-methylation of *PPARγ* in fatty liver subjects [17], which suggests a methylation regulatory target for FLHS. In previous reports, we have discussed the status of lipid metabolism pathways and related genes [3], but methylation analysis was not performed. In this study, we found most genes (e.g., *ACACA*, *APOA4*, and *SCD*) enriched in lipid related pathways have hypo-methylated DMRs and are up-regulated in FLHS. These results suggest a global elevation of lipid biosynthesis, transport, and oxygenolysis to be regulated by methylation network. Furthermore, anabolic pathways, especially the lipogenesis process, dominated the pathological process of FLHS, which is consistent with Liu’s study [16]. Which indicates the methylation changes on lipid metabolism could be a major cause for FLHS.

## Conclusions

In conclusion, our study closely links methylation to chicken FLHS. By integrative analysis, a genome-wide hypo-methylation pattern for FLHS was constructed. The pattern had the following attributes: mRNA expression of genes was inversely correlated with methylation levels for promoters and gene bodies; hypo-methylated and up-regulated genes were mainly enriched in lipid-related pathways (e.g., fatty acid metabolism, PPAR signaling pathway, and fatty acid biosynthesis); by contrast, hyper-methylated and down-regulated genes were mainly enriched in the cellular junction and communication related pathways (e.g., gap junction, phosphatidylinositol signaling pathway, and vascular smooth muscle contraction). These results strongly suggest that increased lipid accumulation and hepatocyte rupture are central pathways that are regulated by DNA methylation in chickens with FLHS.

## Methods

### Ethical statement

All chickens were obtained from the Institute of Animal Sciences, Chinese Academy of Agricultural Sciences (IAS-CAAS, Beijing, China). Ethical approval (reference number: IASCAAS-AE-03) was conferred by the animal ethics committee of IAS-CAAS, which is responsible for animal welfare. All experimental protocols were conducted in accordance with guidelines established by the Ministry of Science and Technology (Beijing, China).

### Animals

The fatty liver susceptible line and control line of Jingxing-Huang chicken were used for experiments [3]. Briefly, for the fatty liver susceptible line, the initial Jingxing-Huang chickens (F0 generation) were induced by a high-fat diet, while the chickens were fed a basal diet for control line. The occurrence of fatty liver, without dietary induction, in the F1 generation was as high as 41.5% (*n* = 82) in the susceptible line and 18.75% (*n* = 80) in control line. Details were described by Zhang et al. previously [3]. In this study, the F1 generation of the two groups were used and all were fed the basal diet. The basal diet was formulated based on NRC (1994) and NY/T (33–2004). Feed and water were provided ad libitum. All the chickens were raised in three-story step cages (one chicken per cage) using the recommended environmental conditions.

### Sample collection

All chickens (*n* = 82 in fatty liver group, *n* = 80 in control group) in F1 generation were euthanized by carbon dioxide anesthesia and exsanguination by severing the carotid artery at 36th week after hatching. The liver samples were collected, snap-frozen and stored at -80 °C for future methylation analysis. Identification of fatty livers was as described [3]. Phenotypic features are shown in Supplementary Figure S1. Four livers with obvious symptom and four normal livers were selected for high-throughput sequencing. Six out of eight liver samples were consistent with our previous report [3].

### DNA library preparation, whole-genome bisulfite sequencing, quality control and mapping

In F1 generation, male chickens with FLHS in the fatty liver group and non-FLHS chickens in the control group were assessed. Genomic DNA was isolated from liver samples (*n* = 4 per group) using the phenol-chloroform method. The integrity was assessed by agarose gel electrophoresis and the purity was checked using the NanoPhotometer® spectrophotometer (IMPLEN, CA, USA), and the concentration was measured using Qubit® DNA Assay Kit in Qubit® 2.0 Flurometer (Life Technologies, CA, USA). After quality control of DNA, library preparation was conducted [34]. Briefly, a total amount of 5.2 μg genomic DNA and 26 ng lambda DNA were fragmented by sonication to generate fragments of 200–300 bp with Covaris S220 (Covairs, Woburn, MA), followed by end repair and adenylation. Then, cytosine-methylated barcodes were ligated to sonicated DNA fragments as instructions. All the DNA fragments were processed twice with bisulfite using EZ DNA Methylation-GoldTM kit (Zymo Research, Orange, CA), before the resulting single-strand DNA fragments were PCR amplificated using KAPA HiFi HotStart Uracil + ReadyMix (2X). The concentration of DNA library was quantified by Qubit® 2.0 Flurometer (Life Technologies, CA, USA) and quantitative PCR, and insert size was assayed based on Agilent Bioanalyzer 2100 system. Due to one library failed, seven libraries were sequenced with the Illumina HiSeq 2500 platform (Novogene, Beijing, China) with more than 20 G of raw data produced, which were deposited in SRA database (accession number: PRJNA682326). After quality control, clean reads were generated using Trimmomatic 0.36 (parameter: slidingwindow: 4:5, leading: 3, trailing: 3, illuminaclip: 2:30:7) [35]. Before mapping, the reference genome (Gallus 5.0) was bisulfite-converted (C to T and G to A) and indexed with bowtie2 [36]. The clean reads were fully bisulfite-converted (C to T and G to A) and then were mapped to the converted genome using Bismark 0.16.3 software (parameter: -X 700 --dovetail) [37].

### DNA methylation analysis and DMGs detection

Before methylation analysis, the duplication caused by PCR amplification was removed using Bismark 0.16.3 [37]. Methylation levels were calculated using the sliding-window (10 kb) method as described [15]. The sum of methylated and unmethylated read counts in each window were calculated. The methylation level for each window and cytosine site is defined as: ML (C) = reads (mC) / (reads (mC) + reads (umC)). Compared to single methylated cytosine sites, DMRs were more efficient for detection of methylation differences [23]. Therefore, DMRs were identified using DSS software, with spatial correlation and biological replicates considered [38, 39]. The DMRs were divided into three types according to the methylated cytosine types, including mCpG, mCHG, and mCHH. Then, DMGs were defined as genes whose promoter or gene body regions overlapped with a DMR.

### DEGs detection and integrative analysis of DEGs and DMGs

Samples for transcriptome analysis were the same as those for WGBS. Transcriptional data were obtained from the GEO database (accession number: GSE111909). The analysis procedure (quality control, mapping to genome, and assembly) and calculation of primary read count were as described in the Zhang et al. study [3]. Briefly, the clean reads were produced from raw reads after removing the reads with one of the standards: 1) the adapter sequence was detected in read, 2) the percentage of N (unknown base) was more than 10%, 3) low quality read (PHRED score ≤ 20, percentage of low quality bases ≥50%). Then, the clean reads were mapped to the reference genome (Gallus 5.0) using HISAT 2.0.4 software with default parameter [40]. And assembly and gene expression quantification steps were performed using cufflinks 2.1.1 and HTSeq v0.6.1 software with default parameter [41, 42]. In this study, the identification of DEGs was performed by DESeq2 (design = ~ group) [43] with a specific standard: fold change (FC) > 1.5 or FC < 0.67, wald *p*-value < 0.05.

To obtain the global profile of methylation and transcription, all genes were ranked by expression level and divided into none, low, medium, and high groups. Average methylation level of those genes in each group was calculated [21]. A Venn plot was performed with the web-based tool Draw Venn Diagram (http://bioinformatics.psb.ugent.be/webtools/Venn/).

### KEGG pathway enrichment analysis

Pathway enrichment analysis was conducted with the overlapping genes of DMGs and DEGs using KOBAS [44], *p* < 0.05 was set as the threshold for significant enrichment.

### Statistical analysis

SPSS 25.0 (SPSS, Chicago, IL, USA) was used for statistical analysis. Data are shown as mean ± standard error. Comparisons were performed by Student’s t-test. A *P* value < 0.05 (*) and P value < 0.01 (**) implied statistically significant difference and highly significant difference, respectively. Graphics were drawn using GraphPad Prism 7 (GraphPad Software, San Diego, CA, USA).

## Supplementary Information

**Additional file 1: Supplementary Figure 1.** Apparent feature of fatty liver and normal liver. (a) Phenotype of fatty liver. The liver presented a yellow, hypertrophy, and greasy appearance, some hemorrhagic point were emerged in the hepatic surface. (b) Phenotype of normal liver. The liver presented a dark red and smooth appearance, no hemorrhagic point were discovered.

**Additional file 2: **S**upplementary Figure 2.** Global methylation pattern (mCHG and mCHH) in fatty liver and normal liver. (a-b) Methylation level of specific site mCHG and mCHH in gene body, up- and down-stream. (c-d) Methylation level of specific site mCHG and mCHH in various genomic regions. Including promoter, 5′ and 3′ UTR, exon, intron, and repeat region.

**Additional file 3: Supplementary Figure 3.** The methylation level (mCHG and mCHH) of all genes with different transcriptional level in gene body, up- and down-stream. (a-b) The methylation level with specific site mCHG and mCHH of all genes grouped by transcriptional level in fatty liver group. (c-d) The methylation level with specific site mCHG and mCHH of all genes grouped by transcriptional level in control group.

**Additional file 4: Supplementary Table 1**. Identification and annotation of DMRs and DMGs between two groups.

**Additional file 5: Supplementary Table 2**. Identification and annotation of DEGs between two groups.

**Additional file 6: Supplementary Table 3**. Overlapping genes between DMGs and DEGs.

## Data Availability

The data that support the findings in this study are available from corresponding author with reasonable request. RNA-seq data were obtained from the GEO database (accession number: GSE111909), while the raw data of WGBS were deposited in SRA database (accession number: PRJNA682326).

## Abbreviations

FLHS: Fatty liver hemorrhagic syndrome
FLS: Fatty liver syndrome
TG: Triglyceride
DMR: Differentially methylated region
DMG: Differentially methylated gene
DEG: Differentially expressed gene
WGBS: Whole-genome bisulfite sequencing
CpG: Cytosine-phosphate bond-guanine
CHG: Cytosine-adenine, cytosine, or thymine-guanine
CHH: Cytosine-adenine, cytosine, or thymine-adenine, cytosine, or thymine
PPAR: Peroxisome proliferator activated receptor
