# Supplementary figures and images for: Integrated analysis of the methylome and transcriptome of chickens with fatty liver hemorrhagic syndrome
Source: BMC Genomics. 2021 Jan 6;22:8. doi: 10.1186/s12864-020-07305-3 (PMC7789526; doi:10.1186/s12864-020-07305-3)

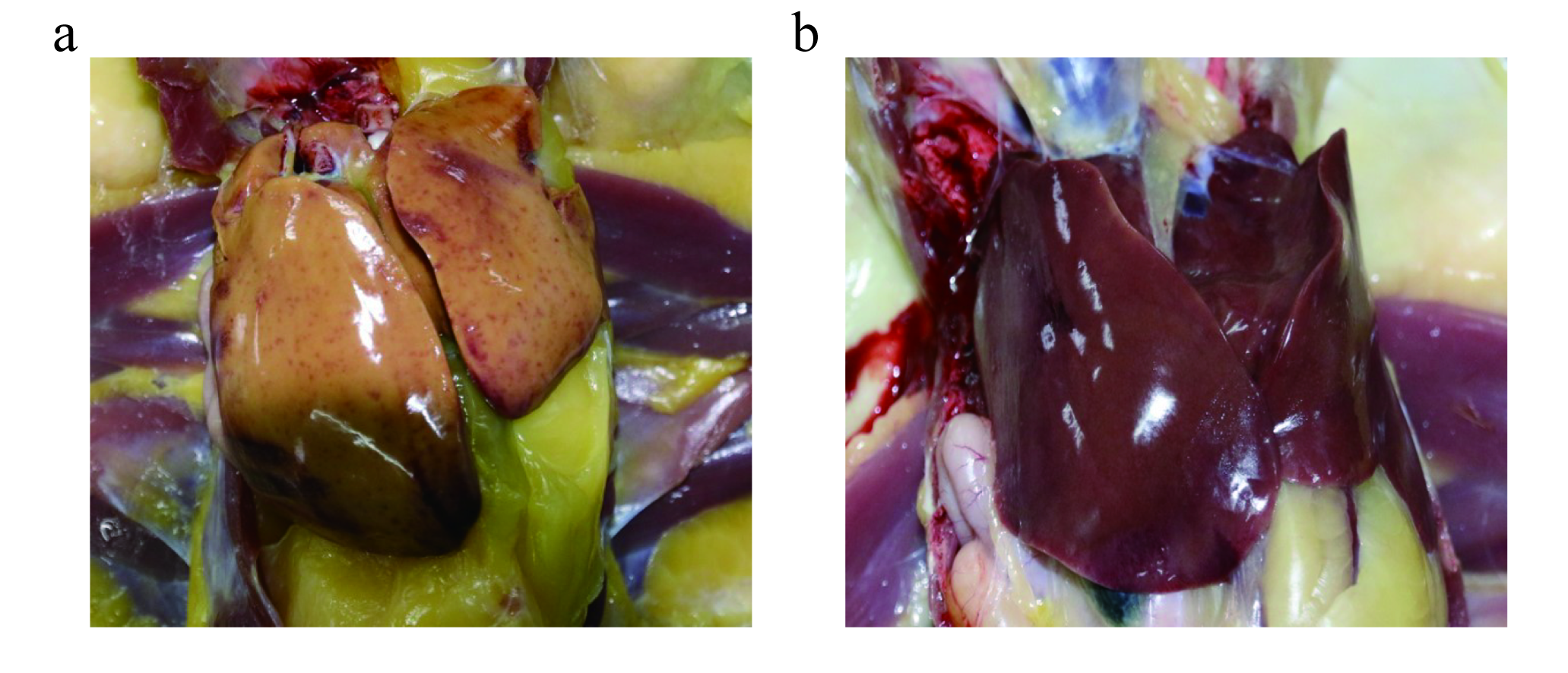

Supplement: Supplementary file 1 — Additional file 1: Supplementary Figure 1. Apparent feature of fatty liver and normal liver. (a) Phenotype of fatty liver. The liver presented a yellow, hypertrophy, and greasy appearance, some hemorrhagic point were emerged in the hepatic surface. (b) Phenotype of normal liver. The liver presented a dark red and smooth appearance, no hemorrhagic point were discovered. [file 12864_2020_7305_MOESM1_ESM.tif]
